# ABHD16A Negatively Regulates the Palmitoylation and Antiviral Function of IFITM Proteins

**DOI:** 10.1128/mbio.02289-22

**Published:** 2022-10-31

**Authors:** Xuemeng Shi, Xiaoling Li, Zhao Xu, Lingyi Shen, Yunyun Ding, Shuaiwu Chen, Lin Mao, Wei Liu, Jun Xu

**Affiliations:** a College of Life Sciences, Henan Agricultural University, Zhengzhou, China; Ohio State University; University of Pennsylvania

**Keywords:** IFITM proteins, antiviral effect, S-palmitoylation, ABHD16A, depalmitoylase, JEV

## Abstract

Interferon-inducible transmembrane (IFITM) proteins are small homologous proteins that are encoded by the interferon-stimulated genes (ISGs), which can be strongly induced by interferon (IFN) and provide resistance to invasion by a variety of viral pathogens. However, the exact molecular mechanisms underlying this function have remained elusive. The antiviral activity of IFITMs from different species depends on S-palmitoylation at conserved cysteine residues. However, specific enzymes involved in the dynamic palmitoylation cycle of IFITMs, especially depalmitoylase, have not yet been reported. Here, we demonstrate that α/-hydrolase domain-containing 16A (ABHD16A) is a depalmitoylase and a negative regulator of IFITM protein that can catalyze the depalmitoyl reaction of S-palmitoylated IFITM proteins, thereby decreasing their antiviral activities on RNA viruses. Using the acyl-PEGyl exchange gel shift (APEGS) assay, we identified ABHD16A proteins from humans, pigs, and mice that can directly participate in the palmitoylation/depalmitoylation cycles of IFITMs in the constructed *abhd16a*^−/−^ cells and ABHD16A-overexpressing cells. Furthermore, we showed that ABHD16A functions as a regulator of subcellular localization of IFITM proteins and is related to the immune system. It is tempting to suggest that pharmacological intervention in IFITMs and ABHD16A can be achieved either through controlling their expression or regulating their activity, thereby providing a broad-spectrum therapeutic strategy for animal viral diseases.

## INTRODUCTION

The innate immune response to viral pathogens relies on various proteins encoded by a series of interferon (IFN)-stimulated genes. Numerous studies have identified IFITM1, IFITM2, and IFITM3 from different species as antiviral restriction factors against multiple enveloped viruses (1–3). *In vitro* studies have highlighted that the restriction by IFITMs occurs at the virus entry stage, which suggests that IFITMs are membrane remodelers because their overexpression may change the physical aspects, positive curvature, and tight junction complexes (4–6). Although the principal molecular mechanism is not completely understood, increasing evidence supports that the IFITM-mediated inhibition of virus production is closely related to interference with membrane fusion (4, 5, 7–10, 11). Importantly, several groups, including ours, have shown that the S-palmitoylation of IFITM is crucial for its antiviral property (12–16). In cells, the process of defending against viruses is dynamic and complex, in which a set of host factors are used. However, to date, only a few proteins that may function in the antiviral response have been identified as IFITM-interacting molecules (3). Moreover, little is known about the special enzyme in the dynamic cycle of IFITM S-palmitoylation (i.e., depalmitoylase).

Palmitoylation/depalmitoylation is an active cycle that acts as a critical step for regulating protein trafficking, localization, stability, and interaction with other proteins in cells (17, 18). Recently, we summarized the palmitoylation modification of both the virus and host cell protein during infection (19). Compared to over 20 palmitoylases, only a few depalmitoylases have been identified to catalyze the S-depalmitoylation reaction. These include acyl protein thioesterase 1 (APT1) and 2 (APT2) (20, 21) and the protein palmitoyl thioesterases 1 (PPT1) and 2 (PPT2) (22–24). Interestingly, ABHD10 and ABHD17, two members of the α/β-hydrolase domain-containing (ABHD) family, have been identified as the members of APTs, respectively. They participate in the S-palmitoylation of peroxiredoxin-5 (PRDX5) (25), Ras-family GTPases, synaptic proteins, and postsynaptic density-95 (PSD-95) (26). Human ABHD16A, also known as human leukocyte antigen B (HLA-B)-associated transcript 5 (BAT5), is located on chromosome 6p21.33 within the human major histocompatibility complex class III (MHC-III) region (27). An analysis using a high-throughput yeast two-hybrid (Y2H) screen targeting the genes of the human MHC-III region showed that ABHD16A and IFITM1 were interacting partners (28). However, whether their interaction affects the function and the related molecular mechanism remains to be explored. ABHD16A is a highly conserved protein and expressed in cells of multiple species. In recent decades, only a few satisfying results on its activities and physiological functions have been reported, presenting certain clues about its functions (29–32). In 2018, we systematically reviewed almost all members of the ABHD family and suggested that ABHD16A might play key roles in the interaction between virus and host cells, signaling pathways, the occurrence and development of diseases, especially from the perspective of specific enzyme activities and immune responses (27). Based on the above findings, we speculated that ABHD16A could play a role in the antiviral effect of IFITMs. Here, we demonstrated ABHD16A is a critical S-depalmitoylase of the palmitoylation/depalmitoylation cycle in human, pig, and mouse IFITMs and regulates their cellular localization and antiviral functions. Our findings provide new insights into the antiviral mechanism of IFITM proteins and indicate that ABHD16A may contribute to the prevention and control of viral infections by pharmacological or genetic disruption or activation.

## RESULTS

### ABHD16A interacts with IFITMs.

**FIG 1:**
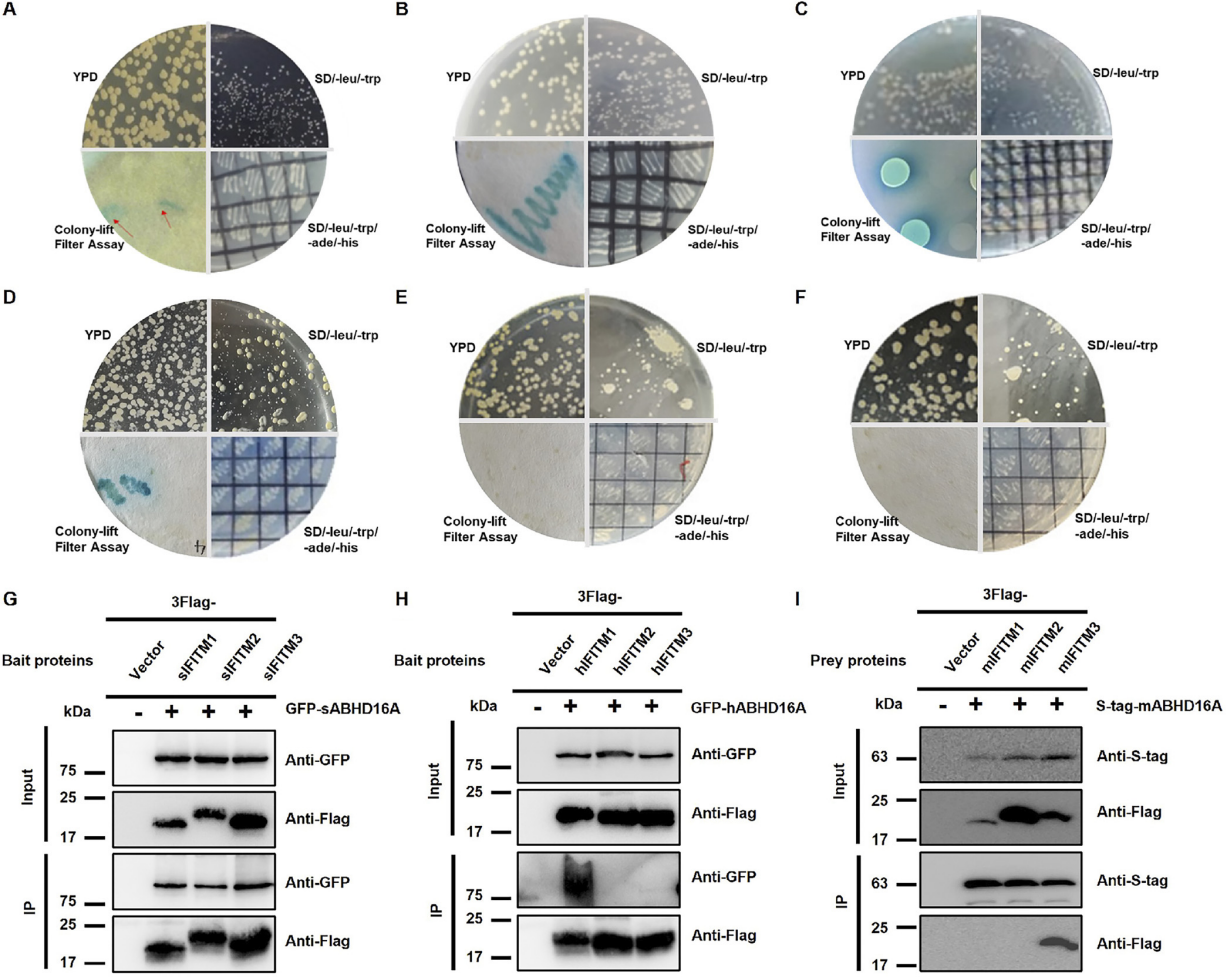
Yeast two-hybrid (Y2H) and co-IP assays to detect the interaction of ABHD16A with IFITMs. In the Y2H assay, the plasmids listed below were cotransfected into AH109 yeast cells. Cells were then cultivated in different selection-deficient culture media. Finally, the color reaction was performed to identify the interaction. Plasmids: pGBKT7-*sifitm1*/pGADT7-*sabhd16a* (A), pGBKT7-*sifitm2*/pGADT7-*sabhd16a* (B), pGBKT7-*sifitm3*/pGADT7-*sabhd16a* (C), pGBKT7-*hifitm1*/pGADT7-*habhd16a* (D), pGBKT7-*hifitm2*/pGADT7-*habhd16a* (E), and pGBKT7-*hifitm3*/pGADT7-*habhd16a* (F). (G) PK15 cells were cotransfected with 3×Flag-sIFITM1/2/3 together with GFP-sABHD16A for 24 h, and cell lysates were immunoprecipitated with anti-Flag antibody. Expression of proteins from the transfected plasmids was analyzed using co-IP and immunoblotting (IB) assays with anti-GFP and anti-Flag antibody, respectively. (H) The interaction between hABHD16A and hIFITM1 was preliminarily confirmed by co-IP assay in HEK293 cells; anti-Flag was used as bait antibody for IP. (I) Expression constructs of S-tag-mABHD16A with empty vector, 3×Flag-mIFITM1, 3×Flag-mIFITM2, or 3×Flag-mIFITM3 were transfected into HEK293 cells and at 24 h posttransfection for immunoprecipitation. The co-IP assay using S-tag-mABHD16A as the bait protein demonstrated the interaction of mIFITM3 with mABHD16A.

To explore the interaction between ABHD16A and IFITMs, we successively adopted the yeast two-hybrid (Y2H) assay and protein coimmunoprecipitation (co-IP) assay. The cotransformed yeast with swine IFITMs (sIFITMs) and ABHD16A not only grew well on SD/−Leu/−Trp/−Ade/−His medium but also showed blue clones, proving that sIFITM1, sIFITM2, and sIFITM3 interact with swine ABHD16A (sABHD16A) (Fig. 1A to C). Since the sequences of IFITM proteins and ABHD16A are highly conserved in different species (27), we next studied the interaction of human IFITM (hIFITM) proteins and ABHD16A using the same Y2H system. Results showed that hABHD16A and hIFITM1 are also interaction proteins, whereas no interaction was observed between hABHD16A and hIFITM2 or hIFITM3 (Fig. 1D to F). Next, we performed co-IP and observed swine ABHD16A was bound to three IFITM proteins—sIFITM1, sIFITM2, and sIFITM3 (Fig. 1G). In contrast, only the interaction between human IFITM1 and ABHD16A was detected (Fig. 1H). In addition, we also explored the relationship between ABHD16A and IFITMs in the mouse by using co-IP *in vitro* and a bimolecular fluorescence complementation (BiFC) system in living cells (33), which indicates mouse IFITM3 (mIFITM3) rather than mIFITM1/2 interacts with mABHD16A (Fig. 1I and see Fig. S1 in the supplemental material). Collectively, these results suggest the interplay of ABHD16A with specific IFITMs among different species.

10.1128/mbio.02289-22.1 FIG S1 Interaction between mABHD16A and mIFITM3. Shown are results from the BiFC assay for detection of the interaction between mIFITM1/2/3 and mABHD16A. HEK293 cells were transiently transfected with plasmids: VN+VC or mABHD16A-VN+mIFITM1/2/3-VC. Twenty-four hours later, cell nucleus was stained with Hoechst 33342 for 10 min. Venus signals denote the interaction in living cells. Scale bar, 20 μm. VN, aa 1 to 173 of Venus; VC, aa 174 to 239 of Venus. Download FIG S1, TIF file, 0.6 MB. Copyright © 2022 Shi et al. 2022 Shi et al. https://creativecommons.org/licenses/by/4.0/ This content is distributed under the terms of the Creative Commons Attribution 4.0 International license.

### ABHD16A plays essential roles in IFITM traffic from the plasma membrane to cytoplasm.

**FIG 2:**
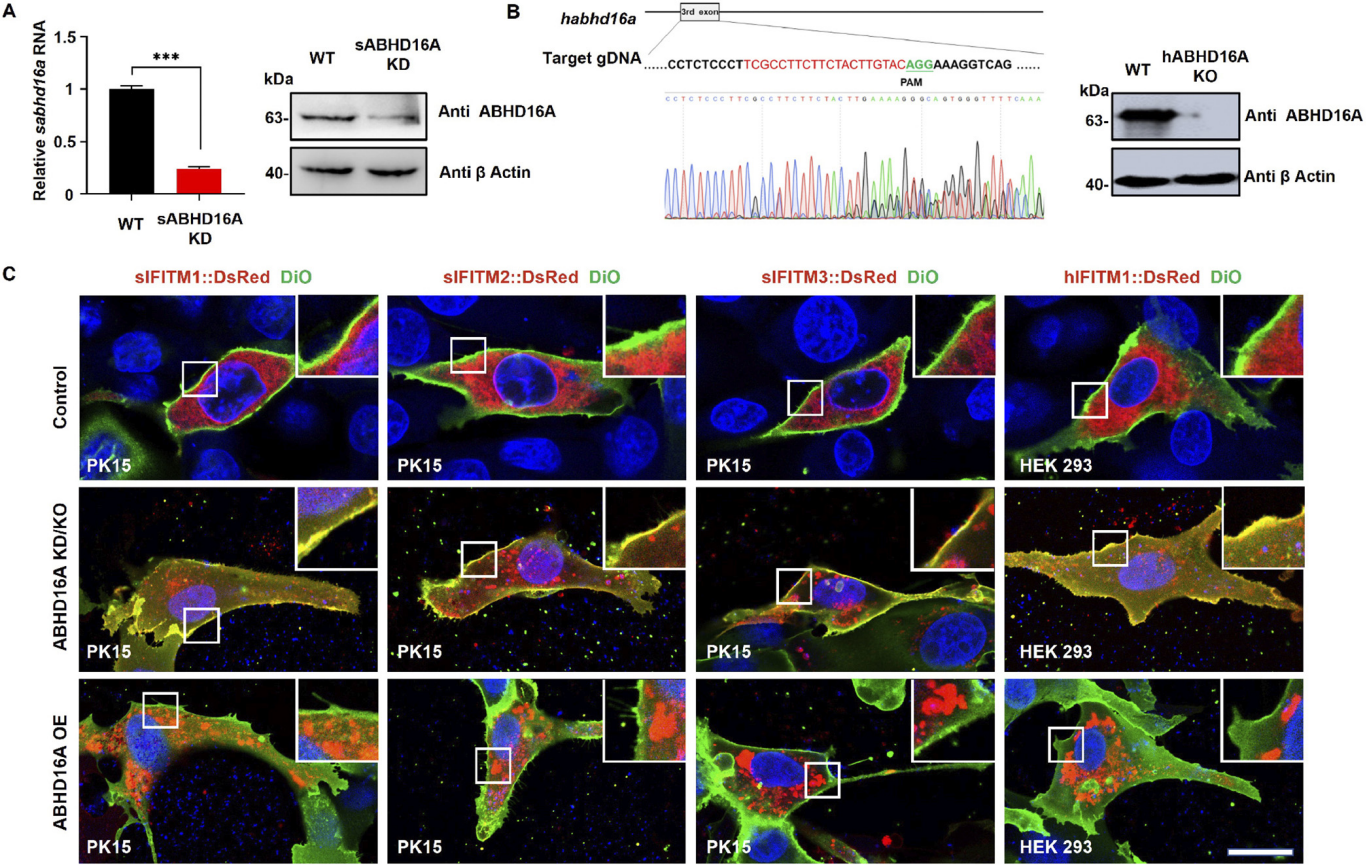
ABHD16A plays essential roles in IFITM trafficking from the plasma membrane to cytoplasm. (A) PK15 cells were transfected with s*abhd16a* shRNA for 48 h, and the mRNA as well as protein levels of sABHD16A were quantified through qRT-PCR and Western blotting, respectively. (B) Sequencing and Western blotting identification of hABHD16A deletion. A set of spikes indicates that h*abhd16a* sgRNA has the phenomenon of editing the genome (gDNA). (C) PK15 cells were cotransfected with expression plasmids of DsRed-sIFITM1/2/3 with shsABHD16A or Flag-sABHD16A, respectively. HEK293 WT and hABHD16A KO cells were transfected with DsRed-hIFITM1 and Flag-hABHD16A, respectively. Forty-eight hours later, cells were fixed with 4% paraformaldehyde, and the plasma membrane and nucleus were stained with DiO and Hoechst 33342 for 15 min, respectively. The insets are magnified views of the boxed areas. KD, knockdown; KO, knockout; OE, overexpression. Scale bar, 10 μm.

To explore the cellular localization of ABHD16A and IFITM proteins, IFITMs and ABHD16A were cotransfected into HEK293 cells. Confocal microscopy imaging demonstrated that all three swine IFITM proteins sIFITM1 to -3 were colocalized with ABHD16A. Pearson's correlation coefficients (*r*) in the scatter plot were greater than 0.9 (Fig. S2A). Human IFITM1 and ABHD16A showed colocalization, whereas partial colocalization was observed for hFITM2 and -3 and hABHD16A (Fig. S2B). These results were consistent with those of the *in vitro* interaction assay (Fig. 1D to F and Fig. 1H), which further verified the interacting relationship between IFITM and ABHD16A but not those of hFITM2 and hFITM3. Next, we investigated whether ABHD16A regulates the subcellar distribution of IFITM proteins by using high-resolution laser scanning confocal microscopy and genomic editing methods in swine and human cells, respectively. PK15 cells were transfected with short hairpin RNA (shRNA) targeting sABHD16A, and by using quantitative real-time PCR (qRT-PCR) and Western blotting (WB), we found the expression of sABHD16A significantly decreased compared with that of control cells (Fig. 2A). Next, we cotransfected DsRed-fused sIFITM1/2/3 with shsABHD16A and observed knockdown (KD) of sABHD16A promotes sIFITM1/2/3 localized on the DiO-labeled plasma membrane (Fig. 2C). In contrast, overexpression (OE) of Flag-sABHD16A results in accumulation of either sIFITM1, sIFITM2, or sIFITM3 in cytoplasm (Fig. 2C). The loss of hABHD16A in HEK293 cells using CRISPR/Cas9 genome editing (Fig. 2B) leads to hIFITM1 being distributed on the plasma membrane, whereas expression of hABHD16A results in cytoplasmic accumulation of hIFITM1 (Fig. 2C). Taken together, we think that ABHD16A plays essential roles in IFITM traffic from the plasma membrane to cytoplasm.

10.1128/mbio.02289-22.2 FIG S2 Subcellular localization of ABHD16A and IFITMs. Shown are results from confocal microscopy imaging of HEK293 cells transiently expressing ABHD16A and IFITM fused with GFP or DsRed, respectively. (A) sIFITM1/2/3 and sABHD16A; (B) hIFITM1/2/3, and hABHD16A. The color gradient on the right side of the scatter plot represents the gray value of each pixel. The abscissa and ordinate, respectively, represent the discrete values of each pixel in the red and green fluorescent images and vary from 0 to 128. *r* refers to Pearson’s correlation coefficient. Hoechst 33342 staining was used to visualize nuclei. White dashed lines indicate the cell outline. The scale bar represents 10 μm. Download FIG S2, TIF file, 0.6 MB. Copyright © 2022 Shi et al. 2022 Shi et al. https://creativecommons.org/licenses/by/4.0/ This content is distributed under the terms of the Creative Commons Attribution 4.0 International license.

### ABHD16A catalyzes the depalmitoylation of IFITMs.

**FIG 3:**
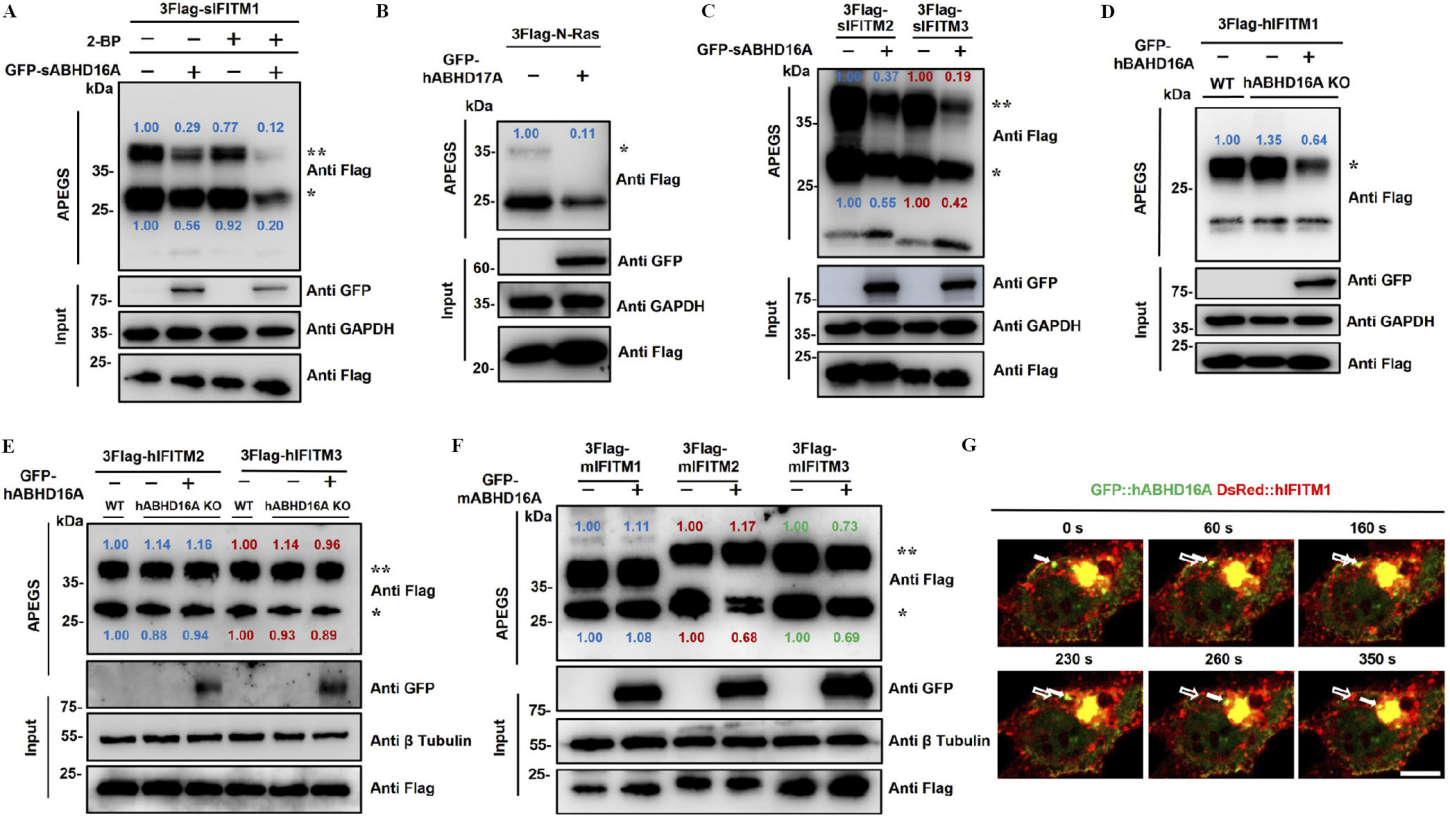
APEGS assay for ABHD16A catalyzing the S-depalmitoylation of IFITMs. HEK293 cells are cotransfected with expression plasmids of ABHD16A and IFITMs, and 36 h later, cell lysates are prepared and incubated with TCEP. Free cysteine residues are protected with NEM. S-fatty acid groups of IFITMs are replaced by 5-kDa mPEG-Mal through two subsequent reactions; a reduction mediated by NH_2_OH results in cysteine exposure and a ligation reaction with mPEG-Mal. Proteins are subjected to SDS-PAGE and analyzed by Western blotting. The number of PEGylation exchanges is indicated by asterisks (*). The relative gray values of the bands (palmitoylated/input) were calculated by ImageJ gel analyzer. (A) sIFITM1; (B) N-Ras; (C) sIFITM2 and sIFITM3; (D) hIFITM1; (E) hIFITM2 and hIFITM3; (F) mIFITM1, mIFITM2, and mIFITM3. (G) Time-lapse imaging of HEK293 cells coexpressing GFP::hABHD16A and DsRed::hIFITM1 reveals that hABHD16A-associated hIFITM1 vesicles move away from plasma membrane. Solid and hollow arrows indicate the starting and ending positions of discrete hABHD16A and hIFITM1 signals, respectively. The recording was set as every 10 s for 360 s. Bar, 10 μm.

How does ABHD16A regulate the subcellular distribution of IFITMs? Based on the interaction of ABHD16A and IFITMs and the functions of the ABHD family reported, as well as the critical role of palmitoylation in protein subcellular localization, we next evaluated whether the interaction of ABHD16A and IFITM was related to the palmitoylation cycles of the IFITM protein. The acyl-PEGyl exchange gel-shift (APEGS) assay is a novel method in which palmitoylated proteins were labeled with methoxy polyethylene glycol maleimide (mPEG-Mal) and detected by Western blotting (16, 19). First, we detected the palmitoylation state of swine IFITM1 using the APEGS assay by single transfection or cotransfection with the expression plasmids of sABHD16A and sIFITM1 into HEK293 cells. The results showed that the mPEG-Mal-linking bands, which represented the palmitoylated sIFITM1, receded by overexpression of sABHD16A (Fig. 3A). The palmitoylation of sIFITM1 was more greatly decreased under the dual effects of sABHD16A and 2-BP, an inhibitor of palmitoyl acyltransferase (34) (Fig. 3A). In addition, we adopted KC01, the specific inhibitor of ABHD16A (31), to check the palmitoylation level of IFITMs. By using APEGS, we observed the palmitoylation of swine IFITM1 increased upon treatment with KC01 (Fig. S3A and B), suggesting that ABHD16A could be a depalmitoylase for sIFITM1. To validate the results of the APEGS assay in depalmitoylase, we used ABHD17A, an identified S-depalmitoylase (26), and one of its substrates, N-Ras protein, as the positive control (Fig. 3B). Further results demonstrated that sABHD16A dramatically reduced the palmitoylation of sIFITM2 and sIFITM3 (Fig. 3C). APEGS analysis preliminarily revealed that sABHD16A may have the activity of S-depalmitoylase—at least for sIFITM1, sIFITM2, and sIFITM3.

10.1128/mbio.02289-22.3 FIG S3 Treatment with ABHD16A inhibitor KC01 increases the palmitoylation of sIFITM1 and inhibits the infection of JEV. (A) The production of lyso-PS was measured by ELISA in KC01-treated control and sABHD16A-overexpressed PK15 cells. (B) APEGS assay for sABHD16A catalyzing the S-depalmitoylation of sIFITM1 in KC01-treated cells. The stably overexpressed sABHD16A PK15 cells are cotransfected with expression plasmids of 3×Flag-sIFITM1 and treated with 5 μM KC01 24 h later, and then cell lysates are prepared and incubated with TCEP. Free cysteine residues are protected with NEM. S-fatty acid groups of sIFITM1 are replaced by 5-kDa mPEG-Mal through two subsequent reactions: a reduction mediated by NH_2_OH that results in cysteine exposure and a ligation reaction with mPEG-Mal. Proteins are subjected to SDS-PAGE and analyzed by Western blotting. The number of PEGylation exchanges is indicated by asterisks. The relative gray values of the bands (palmitoylated/input) were calculated by ImageJ gel analyzer. (C) Intracellular JEV-E mRNA in infected PK15 cells was measured by qRT-PCR 48 h postinfection. Cells were pretreated with KC01 for 24 h before infection. Data are mean ± SEM from three independent experiments. ns, not significant; *, *P* < 0.05; **, *P* < 0.01; ***, *P* < 0.001 (*t* test in panel A and one-way ANOVA in panel C). Download FIG S3, TIF file, 0.4 MB. Copyright © 2022 Shi et al. 2022 Shi et al. https://creativecommons.org/licenses/by/4.0/ This content is distributed under the terms of the Creative Commons Attribution 4.0 International license.

Due to the high homology of the ABHD16A protein in different species (27), we next studied the depalmitoylation effect of human ABHD16A on IFITM proteins in h*abhd16a*^−/−^ HEK293 cells. When the expression plasmid of hIFITM1 was transfected into h*abhd16a*^−/−^ cells or wild-type HEK293 cells, the palmitoylation level of hIFITM1 was higher under hABHD16A deficiency (Fig. 3D). In addition, the bands of palmitoylated hIFITM1 were considerably weaker when hABHD16A was recovered by transfection with hABHD16A expression plasmids (Fig. 3D). Subsequently, we investigated whether hABHD16A catalyzed the depalmitoylation of human IFITM2 and IFITM3. The results indicated that ABHD16A exerted no effect on hIFITM2 and hIFITM3 palmitoylation (Fig. 3E). These data verified that human ABHD16A functions as an S-depalmitoylase for hIFITM1, but not for hIFITM2 and hIFITM3. We further confirmed that mouse ABHD16A lowered the palmitoylation level of mIFITM2 and mIFITM3; however, no significant effect was observed on mIFITM1 (Fig. 3F). Therefore, the palmitoylation analysis shows that ABHD16A of humans, pigs, and mice is direct participant in the palmitoylation/depalmitoylation cycle of specific IFITM proteins.

To further elucidate the detailed dynamics of ABHD16A regulating the palmitoylation/depalmitoylation cycle of IFITM, we conducted a time-lapse experiment by using a live-cell imaging system and revealed hABHD16A-green fluorescent protein (GFP) transports together with DsRed-fused hIFITM1 away from plasma membranes, followed by fusion with the cytoplasmic aggregated signal (Fig. 3G; and see Movie S1 in the supplemental material). Consistent with the results in Fig. 2 and 3, live-cell imaging indicates that ABHD16A catalyzes the depalmitoylation of IFITMs.

10.1128/mbio.02289-22.6 MOVIE S1 ABHD16A traffics IFITM1 away from cellular membranes. Shown is time-lapse imaging of HEK293 cells coexpressing GFP-hABHD16A and DsRed-hIFITM1 revealing that hABHD16A-associated hIFITM1 vesicles move away from plasma membrane. Solid and hollow arrows indicate the starting and ending position of discrete hABHD16A and hIFITM1 signals, respectively. The recording was set as every 10 s for 360 s. Bar, 10 μm. Playback is 8 fps. Download Movie S1, AVI file, 12.3 MB. Copyright © 2022 Shi et al. 2022 Shi et al. https://creativecommons.org/licenses/by/4.0/ This content is distributed under the terms of the Creative Commons Attribution 4.0 International license.

### Identification of key catalytic sites or motifs of sABHD16A.

**FIG 4:**
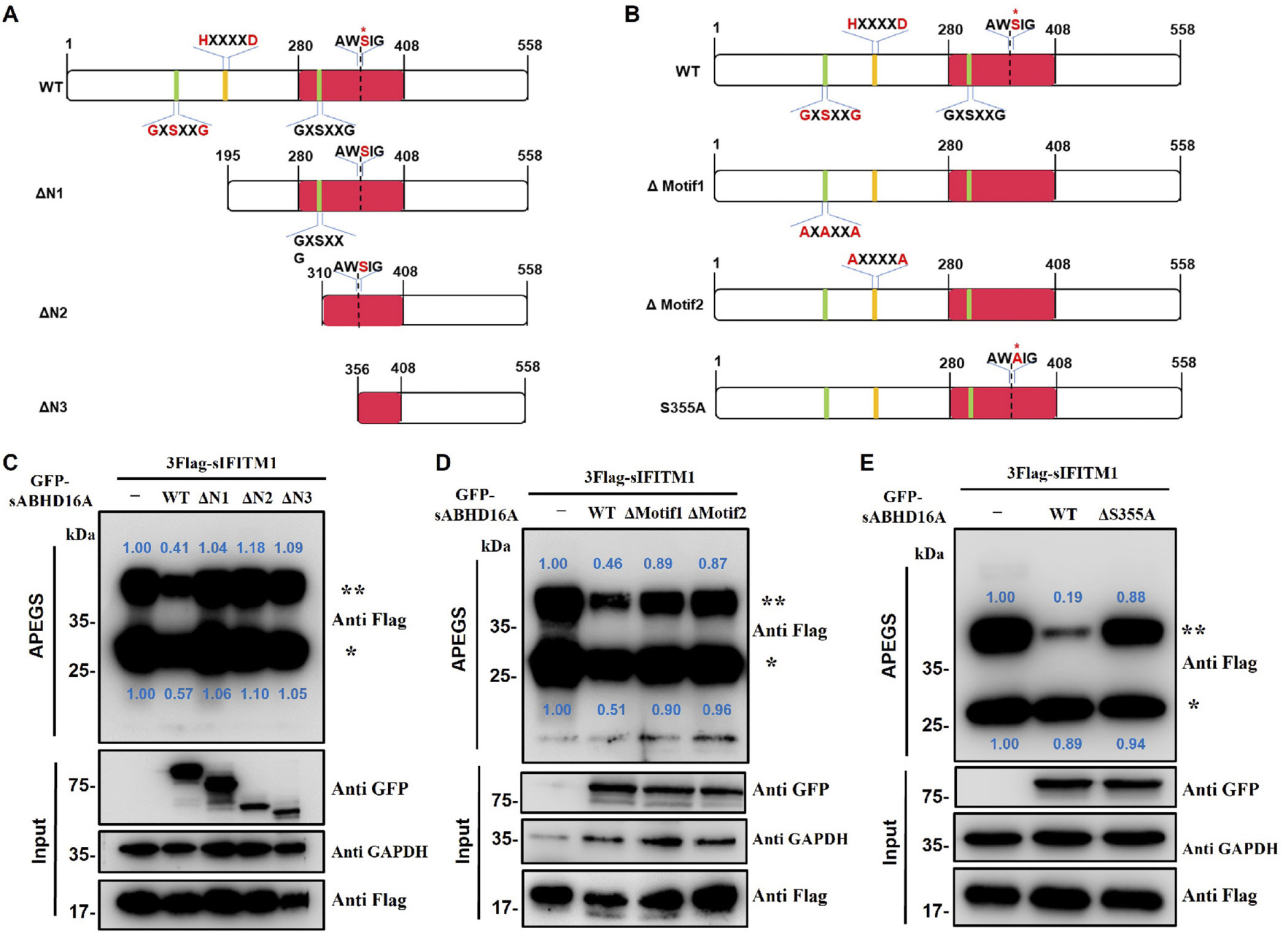
Identification of key sites or motifs of sABHD16A. Schematic for three N-terminally truncated mutants (A) and point mutants (B) of sABHD16A. Rose-red box, α/β-hydrolase domain; green box, lipase-like motifs; yellow box, acyltransferase motif; red asterisk, the predicted catalytic site. The effects of sABHD16A mutants, including three N-terminally truncated mutants (C), two motif mutants (D), and (E) the predicted sites of ABHD16A on sIFITM1 palmitoylation, were monitored through the APEGS assay. The number of PEGylation exchanges is indicated by asterisks. The relative gray values of the bands (palmitoylated/input) were calculated by ImageJ gel analyzer.

To determine which amino acids or motifs play essential roles in the depalmitoylase activity of sABHD16A, we constructed a series of mutants: namely the ΔN1, ΔN2, and ΔN3 mutants, as N-terminally truncated mutants; the Δmotif1 and Δmotif2 mutants, which target its two important N-terminal motifs, the lipase-like motif (GXSXXG) and acyltransferase motif (HXXXXD); and the S355A site mutant (Ser at position 355 was predicted as the active site) (27) (Fig. 4A and B). The APEG assay demonstrated that, compared with the wild-type sABHD16A, the expression of three different truncation mutants of sABHD16A increased the mPEG-Mal-linking bands of sIFITM1, indicating that the critical motifs for depalmitoylase activity may be located at its N terminus (Fig. 4C). Similar effects were observed when S355, GXSXXG, and HXXXXD were mutated by site-directed methods (Fig. 4D and E). Therefore, these mutants revealed that N terminus, especially motifs GXSXXG and HXXXXD, are essential for ABHD16A function. Moreover, S355 is an essential amino acid to remove the palmitoyl groups in its substrates.

**FIG 5:**
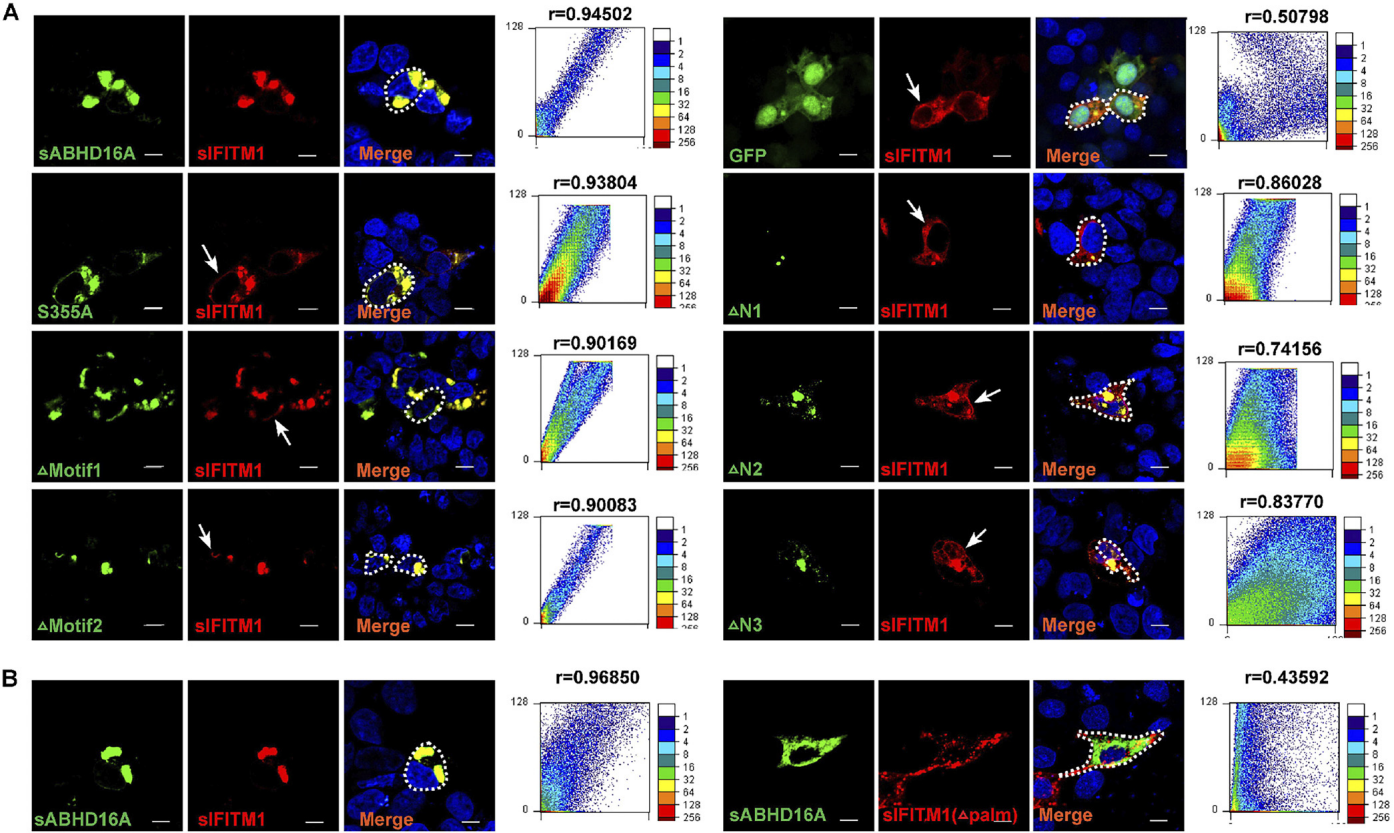
Subcellular localization of sIFITM1 following expression of sABHD16A and its mutants. HEK293 cells were cotransfected with recombinant plasmids of DsRed-sIFITM1 and series of mutants, including the sABHD16A ΔMotif1, ΔMotif2, S355A, ΔN1, ΔN2, and ΔN3 mutants with GFP tag. After 24 h, confocal microscopy imaging of sIFITM1 and sABHD16A was performed. (B) PK15 cells were cotransfected with Δpalm mutants of sIFITM and wild-type sABHD16A. Hoechst 33342 staining was used to visualize nuclei. The scale bar represents 10 μm. The color gradient on the right side of the scatter plot represents the gray value of each pixel. The abscissa and ordinate, respectively, represent the discrete values of each pixel in the red and green fluorescent images and vary from 0 to 128. *r* is the Pearson’s correlation coefficient. Dashed lines indicate the cell outline, and arrows denote the plasma membrane-localized sIFITM1.

To identify the relationship between the enzyme activity and its cellular distribution, we further assessed whether sABHD16A mutants affected the distribution of sIFITM1. The confocal microscopy images showed that the S355A mutant and the two motif mutants did not affect the colocalization of sABHD16A and sIFITM1. However, the fluorescence of colocalization on the cell membrane is enhanced significantly compared with the wild-type sABHD16A (Fig. 5A and Fig. S2A). Three truncated mutants of ABHD16A were partially colocalized with sIFITM1, and interestingly, on the plasma membrane, sIFITM1 became more distinct, while ABHD16A was unobservable (Fig. 5A). Thus, the N terminus of 195 amino acids, not just GXSXXG motif, is important not only for regulating the sIFITM1 subcellular position (trafficking) but also for its catalytic activity in the palmitoylation/depalmitoylation cycle. Because palmitoylation modification mainly occurs at the cystine of a target protein, we next investigated the colocalization of two proteins when three cysteines of sIFITM1 were replaced with serines (Δpalm; C505184S). We found that the Δpalm mutant of sIFITM1 did not colocalize with sABHD16A in PK15 cells (Fig. 5B). Altogether, these findings suggested that the critical motif and S355 of ABHD16A and cysteines of sIFITM1 play important roles in their subcellular localization and functions.

### ABHD16A negatively regulates the antiviral activity of IFITM.

**FIG 6:**
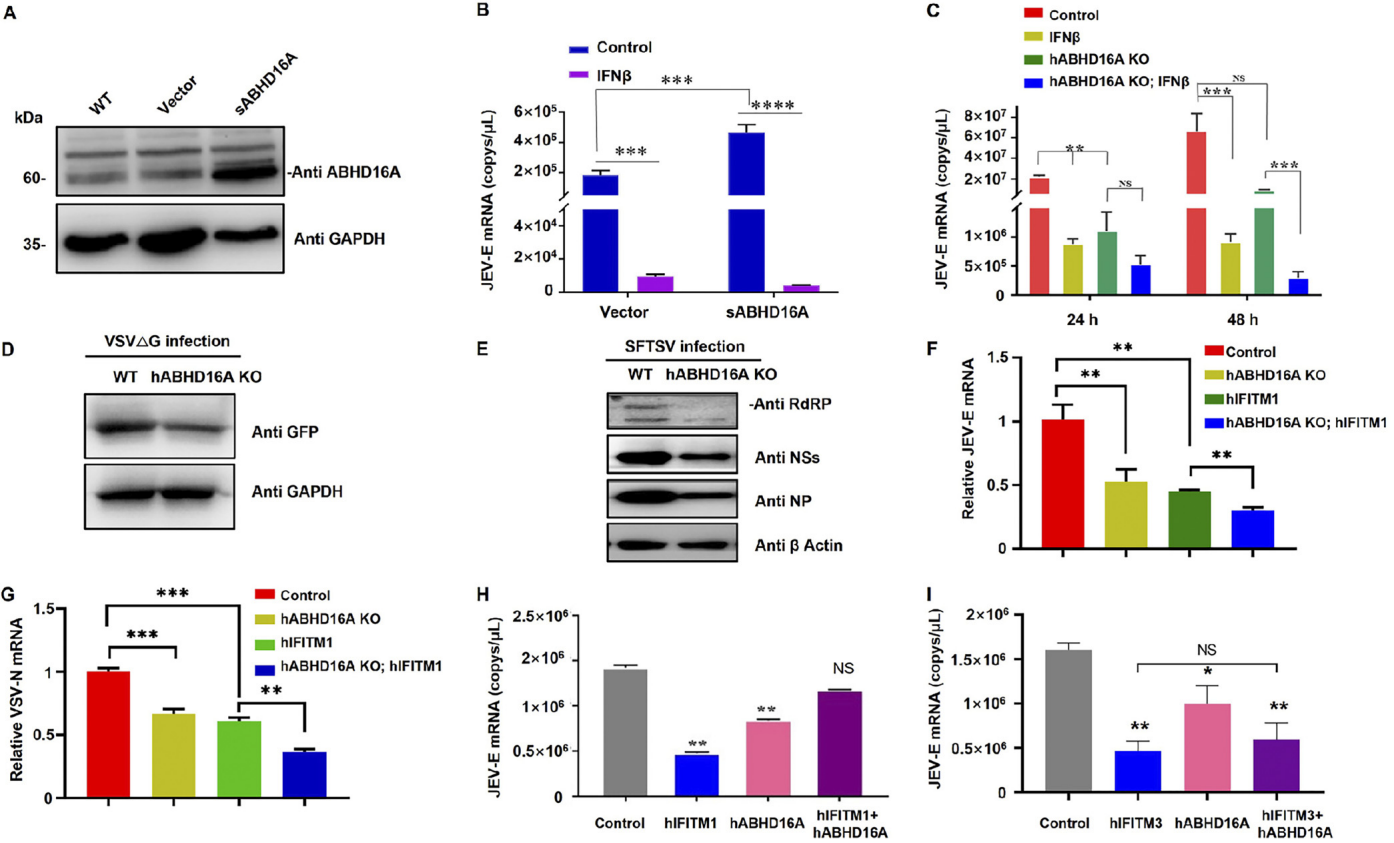
ABHD16A negatively regulates the antiviral activity of IFITM1. (A) Western blotting of PK15 cells stably expressing sABHD16A; (B) qPCR of JEV-E mRNA in the culture medium of PK15 cells stably expressing sABHD16A (PK15-sABHD16A) and empty vector (PK15-vector). IFN-β (500 μU/mL) was used for stimulation for 24 h before cells were infected with JEV SA14-14-2 (MOI = 0.1, 48 hpi) (C) The cell culture supernatants were collected to detect JEV-E mRNA in each the indicated HEK293 cells by qPCR. MOI = 0.1. (D) Intracellular VSVΔG GFP levels in infected WT and hABHD16A KO HEK293 cells (MOI = 0.1) were measured by Western blotting 48 hpi. (E) Intracellular SFTSV RdRP, NSs and NP protein level in infected WT and hABHD16A KO HEK293 cells (MOI = 0.1) were measured by Western blotting 48 hpi. (F and G) Intracellular JEV-E (F) and VSV-N (G) mRNAs in each of the indicated cells were measured by qRT-PCR 4 h after infection. MOI = 0.1. (H and I) qPCR of JEV-E mRNA in the culture medium of HEK293 cells transiently expressing human IFITM1 or hIFITM3 or ABHD16A. Cells were infected with JEV SA14-14-2 (MOI = 0.1, 48 hpi), Data are mean ± SEM from three independent experiments. ns, not significant; *, *P* < 0.05; **, *P* < 0.01; ***, *P* < 0.001 (one-way ANOVA).

Previous studies have confirmed that the palmitoylation of IFITMs is indispensable for its antiviral activity (12, 13, 35, 36), including our study, which revealed that the anti-Japanese encephalitis virus (anti-JEV) activity of swine IFITM proteins was dependent on S-palmitoylation at three cysteine residues (16). In follow-up experiments, we estimated the impact of ABHD16A on the antiviral effects of IFITMs. We constructed a PK15 cell line stably expressing sABHD16A (Fig. 6A) and infected it using the JEV SA14-14-2 strain. The amount of JEV-E mRNA in culture media increased upon expression of sABHD16A (Fig. 6B). Additionally, JEV infection was significantly inhibited following beta interferon (IFN-β) treatment in both cells stably expressing sABHD16A and control cells. The cells stably expressing sABHD16A are likely to be more sensitive to IFN-β (Fig. 6B). In addition, we determined the virus activity when PK15 cells were treated with KC01 and found that this specific inhibitor of ABHD16A induced a dose-dependent reduction in the virus copy number (Fig. S3C). Next, we investigated whether human ABHD16A exerted a similar effect on the anti-JEV activity. The number of JEV copies derived from HEK293 cell culture medium after 24 and 48 h of infection sharply decreased in the absence of hABHD16A expression, and IFN-β stimulation remarkably inhibited the JEV infection (Fig. 6C). Moreover, we adopted another two RNA viruses to reveal the role of ABHD16A in regulating virus infection. Wild-type and hABHD16A knockout (KO) HEK293 cells were infected with thrombocytopenia syndrome virus (SFTSV) and vesicular stomatitis virus ΔG (VSVΔG) pseudotypes (37, 38), respectively. The absence of hABHD16A significantly reduced SFTSV RdRP, NS, NP, and VSVΔG GFP protein levels at 48 hpi, respectively (Fig. 6D and E). Given that IFITMs have been reported to inhibit virus-cell fusion on the plasma membrane (1, 4–6), we were determined to study whether ABHD16A and IFITM respond to infection at the virus entry step. Cells were infected with JEV or VSVΔG pseudotypes for 4 h, washed, and then collected for extraction and measurement of viral mRNA. It is apparent that knockout of hABHD16A causes defective entry of viruses (Fig. 6F and G). Collectively, these data revealed depletion of ABHD16A reduce virus infection.

Subsequently, we explored the impact of ABHD16A on the antiviral effect of IFITM. First, by checking intracellular JEV-E and VSV-N mRNA level, we observed that hABHD16A deficiency promotes hIFITM1 inhibition of virus infection (Fig. 6F and G). Furthermore, by transfection of hABHD16A in HEK293 cells, the JEV-E mRNA in supernatants indicated that ABHD16A counteracted the antiviral activity of IFITM1 rather than IFITM3 (Fig. 6H and I). Therefore, these results suggested that ABHD16A is a negative regulator of a specific IFITM antiviral effect.

## DISCUSSION

The present study demonstrated that ABHD16A is a depalmitoylase targeting IFITM proteins, thereby negatively regulating their antiviral activity. In addition, we established an important role of ABHD16A as antiviral immunoregulatory factor. It is well known that palmitoylation of proteins is an important reversible posttranslational modification (PTM) required for protein-protein interactions, membrane targeting, and intercellular and intracellular signaling. Furthermore, the S-palmitoylation cycle has been proposed as a novel drug target against pathogens due to its wide range of biological effects. Palmitoylation of viral and host proteins during infection has been reported in the majority of eukaryotic permissive cell types (39–41). However, in this dynamic cycle, a more encouraging prospect of depalmitoylation might be uncovered as only a few acyl-protein thioesterases (APTs) have been discovered compared to the dozens of palmitoylases/palmitoyl acyltransferases (PATs) reported. PATs are also named zDHHC enzymes because they contain both Zn^2+^ binding domains and conserved Asp-His-His-Cys (DHHC) motifs (42, 43). The reversible nature of palmitoylation contributes to the dynamic relocalization of proteins between plasma membranes and the cytoplasm. Studies have demonstrated that PTM plays multiple roles in virus assembly or pathogenesis (44–46). As a broad-spectrum antiviral restriction factor in different animals, the S-palmitoylation of conservative cysteine residues of IFITM is essential to inhibit viruses (16, 36, 47, 48). McMichael et al. reported that palmitoylases, such as DHHC20 and ZDHHC1, increased the IFITM3 antiviral activity against influenza virus (49). However, palmitoylation is dispensable for IFITM3 restriction of severe acute respiratory syndrome coronavirus 2 (SARS-CoV-2) infection (8), which may be due to the distinct endocytic transport or fusion kinetics of influenza virus and SARS-CoV-2. It is worth noting that the enzyme that catalyzes the depalmitoylation of IFITMs has not been reported yet. Our results clearly demonstrated an interactive relationship between ABHD16A and IIFTMs (Fig. 1) and further verified ABHD16A as a novel depalmitoylase (Fig. 3) that can regulate the sublocalization and antiviral activity of IFITM (Fig. 2, 5, and 6), thus providing an unexpected insight into the role of ABHD16A in antiviral immunoregulation.

IFITM proteins, as potent restriction factor against multiple viruses, are membrane organizers and alter the membrane properties, thus blocking the membrane fusion of endocytosed viruses (4, 5). However, the accumulation of large amounts of IFITM proteins in the membrane can be harmful to the host during chronic or persistent infection. Therefore, for IFN signaling, an intriguing and still poorly addressed question is how the host regulates the innate immune response in a balanced manner. Here, we have demonstrated a novel function of ABHD16A as a negative regulator of IFITM proteins by participating in their palmitoylation/depalmitoylation cycles, possibly dislocating IFITM from the membrane due to the loss of membranophilic palmitoyl groups. Depalmitoylase activities of ABHD16A from humans, pigs, and mice on IFITMs and its conservation in different species imply that the depalmitoylation of IFITMs might be widely present in other species. This dynamic modulation of IFITM by ABHD16A, to a certain extent, may shed light on the possible engagement of these proteins in the cross talk in the innate immune system. Buchrieser et al. and Zani reported that IFITM proteins promoted fetal demise by inhibiting the formation of placental syncytiotrophoblasts; this inhibition is dependent on the palmitoylation of IFITM (50, 51). Therefore, it will be worth determining whether ABHD16A can inhibit IFN-mediated disorders, such as fetal demise caused by virus infection. Intriguingly, although IFITM proteins have a more widely recognized role as restriction factors affecting many viruses, the opposite results have also been reported. For example, IFITM3 can be used as an entry factor by viruses to facilitate their infection, such as human coronavirus OC43 and emerging SARS-CoV-2 (52). It is most likely that IFITM3 promotes the low-pH-activated membrane fusion that is essential for CoV infection. Considering that not all IFITMs from pigs, humans, and mice can interact with ABHD16A, we do not completely understand the details of how the depalmitoylase activity of ABHD16A changes the cellular localization and affects the antiviral efficiency of different IFITM proteins. Our results provide experimental evidence and shed light on further related mechanical studies. Detailed analysis of whether the interaction between IFITMs and ABHD16A plays a role in viral entry would be helpful in revealing the molecular mechanism of the game between viruses and hosts.

JEV is a mosquito-borne zoonotic flavivirus responsible for acute viral encephalitis in humans and causes boar orchitis, piglet brain inflammation, abortion, and stillbirth (53, 54). However, the molecular mechanisms contributing to the severe pathogenesis, whether in humans or pigs, are poorly understood. We earlier reported that the anti-JEV functions of swine IFITM depend on the palmitoylation of three cysteine residues (16). Here, for the first time, we identified ABHD16A of three different species as protein interacting with IFITM by using Y2H, co-IP, and APEGS assays. Not all IFITM proteins were verified as partners of ABHD16A: for example, murine ABHD16A could not regulate the palmitoylation of murine IFITM1 (Fig. 3F). For JEV replication or infection, ABHD16A functions as a favorable factor by trafficking IFITM away from the membrane by removing the palmitoyl groups of IFITM, which are essential for its anchoring into the lipophilic membrane.

Tissue differential expression analysis in humans, pigs, and mice from several databases, including GeneCard, showed a high expression of ABHD16A in the testis, brain, and reproductive organs. Therefore, it is necessary to explore whether the pathogenesis is related to the high expression of ABHD16A in these tissues. The gene localization of ABHD16A on the highly conservative MHC-III region in pigs, mice, and humans indicates its possible functions in immune responses. Our results shown here suggest that the interaction between IFITM and ABHD16A by depalmitoylation could modulate the immune responses to the virus entry or pathogenesis. Thus, the negative regulation of IFITM antiviral activity through ABHD16A may be an intrinsic mechanism through which organisms can balance the antiviral immune responses to avoid the disorders induced by elevated IFN levels.

In conclusion, our study demonstrated ABHD16A as a negative factor for antiviral effects of IFITM proteins via IFITM depalmitoylation. The highly conserved sequences of ABHD16A in different species suggest that the functions in the innate immunity of ABHD16A may be immanent in different mammals. Our findings imply that the pharmacological intervention in IFITM and ABHD16A, either through controlling their expression or regulating their activities, could provide a broad-spectrum therapeutic strategy for animal viral diseases and complications linked to interferon elevation.

## MATERIALS AND METHODS

### Plasmid construction.

The cDNAs of IFITM1, IFITM2, IFITM3, and ABHD16A from humans, pigs, and mice were synthesized from the isolated total RNA of Homo sapiens embryonic kidney HEK293 cells, porcine kidney epithelial PK15 cells, and mouse fibroblast 3T3 cells, respectively, using specific primers (see Table S1 in the supplemental material). To generate 3×Flag-, MYC-, S-tag-, VN173-, VC174-, DsRed-, and GFP-tagged IFITMs or ABHD16A, the cDNA fragments were digested with EcoRI and BamHI and individually cloned into the same sites of pcDNA3.1(+), pEYFP-N1, pDsRed-monomer-N1 and pEGFP-N1 vector, respectively. To knock down sABHD16A in PK15 cells, swine s*abhd16a* (GeneID 100155979) targeting sequence (5′-CGGCTGGTGGAAGAGTGTAAT-3′ was cloned into pLKO.1-TRC-Puro vector through AgeI and EcoRI sites. The primary vectors were purchased from Invitrogen (CA, USA). Restriction endonucleases, ligases, and other molecular biological reagents were obtained from TaKaRa (Japan).

10.1128/mbio.02289-22.4 TABLE S1 Primers for the construction of different recombinant vectors. Download Table S1, DOCX file, 0.02 MB. Copyright © 2022 Shi et al. 2022 Shi et al. https://creativecommons.org/licenses/by/4.0/ This content is distributed under the terms of the Creative Commons Attribution 4.0 International license.

### Cell culture and transfection.

Porcine kidney PK15 cells, Homo sapiens embryonic kidney HEK293 cells, and HEK293T cells were cultured in Dulbecco's modified Eagle's medium (DMEM) containing 10% fetal calf serum (FCS), 100 U/mL penicillin, 100 μg/mL streptomycin, and 4 mM l-glutamine with 5% CO_2_ at 37°C. Approximately 5 × 10^4^ to 6 × 10^4^ cells were seeded into 24-well plates, and 2 × 10^5^ cells were seeded into 6-well plates. After cells adhered to the plate for 18 to 24 h, the expression plasmids with objective genes and corresponding controls were introduced into cells using the X-treme GENE HP DNA transfection reagent (06366236001; Roche, Switzerland).

### Yeast two-hybrid assay.

The yeast two-hybrid vectors pGADT7 and pGBKT7, media, and reagents were purchased from Clontech (CA, USA). The IFITM cDNAs of human and swine sequences were inserted into the bait vector expressing the Gal4-binding domain (BD)-IFITM fusion protein. The ABHD16A cDNA sequence of humans and swine was cloned into the prey vector with the activation domain (AD) using specific primers (Table S1). The wild yeast colonies were cultured in yeast extract-peptone-dextrose (YPD) medium at 30°C for 3 to 5 days. A single colony was transferred into fluid YPD medium to culture at 30°C at 230 rpm for 16 to 18 h and subsequently used to prepare competent yeast cells. Afterward, the recombinant vectors were transformed into competent yeast cells. The transformed yeast cells were transferred onto synthetic dropout (SD) medium: SD−Leu/−Trp plates for amplification, followed by spreading onto the SD−Leu/−Trp/−Ade medium for screening. Afterward, the yeast colonies were transferred to the SD−Ade/−His/−Leu/−Trp minimal medium. The protein interaction was confirmed by checking the blue colonies using the Z buffer–X-α-Gal (5-bromo-4-chloro-3-indolyl-α-d-galactopyranoside) assay.

### Coimmunoprecipitation.

The expression plasmids with 3×Flag-sIFITM1, 3×Flag-sIFITM2, and 3×Flag-sIFITM3 were coexpressed with GFP-sABHD16A in PK15 cells. To reveal the interaction between hIFITM1/2/3 with hABHD16A, HEK293 cells were cotransfected with 3×Flag-hIFITM1/2/3 with GFP-hABHD16A. To check the interaction between mIFITM1/2/3 with mABHD16A, HEK293 cells were transfected with the expression plasmid of 3×Flag-mIFITM1/2/3 and S-tag-ABHD16A. After 24 h of cultivation, cells were harvested using immune coprecipitation lysis buffer (20 mM Tris [pH 7.5], 150 mM NaCl, 1% Triton X-100, 1% sodium deoxycholate, and 0.1% SDS supplemented with 1 mM phenylmethylsulfonyl fluoride [PMSF], 10 mM dithiothreitol [DTT], 40 μg/mL DNase I and 1 μg/mL leupeptin, pepstatin, and aprotinin). Next, magnetic beads and the monoclonal anti-Flag M2 or anti-S-tag antibody were added to the reaction system and incubated at 4°C for 4 h. Afterward, the magnetic beads were washed with phosphate-buffered saline (PBS) three times and boiled in 1× SDS-PAGE loading buffer for 5 min. The supernatant was subjected to SDS-PAGE followed by Western blotting.

### Confocal microscopy.

The cDNA sequences of IFITMs and ABHD16A were ligated into the fusion expression vectors with GFP or DsRed, respectively, and cotransfected into HEK293 cells; 48 h later, the localization of IFITMs and ABHD16A inside the cell was analyzed by the fluorescence of GFP and DsRed using a Leica TCS SP8 laser scanning confocal microscope with a Nikon Plan Apo ×60/1.5 oil lens objective. To label the plasma membrane, cells were incubated with 5 μM DiO (D4292; Sigma, MO, USA) for 10 min and then washed with PBS twice and immersed in complete DMEM. To analyze the colocalization of two proteins inside the cell, we used ScatterJ, one of the plugins of ImageJ, to display Pearson’s correlation coefficient on a colocalization scatterplot (55).

### Live-cell imaging.

For live-cell imaging, 35-mm glass-bottom dishes (BS-20-GJM, Biosharp, China) were coated with 10 μg/mL fibronectin (F2006; Sigma, MO, USA) in PBS for at least 3 h at 37°C, washed with PBS twice, and immersed in complete DMEM without phenol red (PM150211; Procell, China) before seeding of cells. The time-lapse images of cells with transient transfection of hABHD16A-GFP and hIFITM1-DsRed were acquired with Leica TCS SP8 laser scanning confocal microscope. Appropriate filters, a heated sample environment (37°C), controlled 5% CO_2_, and a ×60/1.5 oil objective were used. The recording was set as every 10 s for 360 s, and one focal plane was recorded for all live cell videos.

### BiFC assay for detecting mABHD16A and mIFITM3 interactions in living cells.

Briefly, we cloned the cDNA fragment of m*abhd16a* and m*ifitm1*/*2*/*3* fused with VN173 (amino acids [aa] 1 to 173 of Venus, a variant of yellow fluorescent protein [YFP], made by mutagenesis of pEYFP-N1 in previous studies) and VC174 (aa 174 to 239 of Venus), respectively (33, 56). HEK293 cells were transiently transfected with the following plasmids: VN+VC or mABHD16A-VN+mIFITM1/2/3-VC. Twenty-four hours later, cell nucleus was stained with Hoechst 33342 for 10 min. Venus signals denote the interaction in living cells.

### Drug treatment.

The following drugs were used at a defined dose and time: 2-BP (238422; Sigma, MO, USA) at 100 μM for 6 h and KC01 (GC12789; GlpBio, CA, USA) at 5 μM for 24 h.

### Estimation of lyso-PS.

The cell culture medium was centrifuged at 1,000 × *g* for 20 min to remove cell debris. For lysophosphatidylserine (lyso-PS) estimation, a solid-phase sandwich enzyme-linked immunosorbent assay (ELISA) kit was used (DB470; Huding Biological, China) according to the manufacturer’s protocol. The concentration of lyso-PS in each well was measured by determining the absorbance at 450 nm and its extrapolation with the standard curve.

### Infection and antiviral assay.

The JEV live vaccine strain SA14-14-2, SFTSV, and VSVΔG pseudotypes were propagated once in Vero cells. Viruses containing the supernatant medium were harvested from day 2 postinfection and stored at −80°C. The mRNA of the JEV and VSVΔG was measured by quantitative real-time PCR (qRT-PCR) using a pair of specific primers targeted at the envelope protein (E) gene and nucleocapsid protein (N), respectively (Table S2). The viruses at multiplicities of infection (MOI) of 0.1 to 1 were added to the medium of cultured cells. After attachment for 1 h, the culture medium was removed, and cells were washed with PBS twice. The infected cells were incubated in a fresh medium containing 2% fetal calf serum (FCS) at 37°C for 4, 24, or 48 h according to different experiments. The culture supernatants and cells were collected at corresponding time points. The RNA of the collected virus from the media and the total cellular RNA were extracted using TRIzol (Invitrogen, MA, USA), and quantitated by qPCR. The cellular proteins were extracted and detected by Western blotting.

10.1128/mbio.02289-22.5 TABLE S2 Primers for qPCR of viral infection experiments. Download Table S2, DOCX file, 0.01 MB. Copyright © 2022 Shi et al. 2022 Shi et al. https://creativecommons.org/licenses/by/4.0/ This content is distributed under the terms of the Creative Commons Attribution 4.0 International license.

### Quantitative real-time PCR.

The extracted RNAs were reverse transcribed to cDNA using SuperScript (Invitrogen, MA, USA). The synthetic cDNAs of sABHD16A, JEV-E, and VSV-N were amplified and measured by qPCR using specific primers (Table S2) and the SYBR green qPCR kit (Vazyme, China). The copy number of JEV was determined using standard curve conversion from the cycle threshold (*C_T_*) of a positive-control plasmid carrying the JEV-E gene sequence. The relative expression of other target genes was quantified using the ΔΔ*C_T_* method. *gapdh* and *β-actin* genes were used as endogenous references.

### Western blotting.

Plates were rinsed twice with PBS before harvesting of the adherent cells. The collected cells were lysed with 1 mL of radioimmunoprecipitation assay (RIPA) lysis buffer (R0010; Solarbio, China) supplemented with 1 mM phenylmethyl sulfonyl fluoride (PMSF), 10 mM dithiothreitol (DTT), 40 μg/mL DNase I, and 1 μg/mL leupeptin, pepstatin, and aprotinin for 30 min on ice. Cell lysis was performed by centrifugation at 12,000 rpm for 30 min at 4°C. The supernatant was collected, and the protein concentration was measured using the bicinchoninic acid (BCA) protein assay kit. Equal amounts of protein were mixed with 1× SDS-PAGE loading buffer, boiled, and subjected to SDS-PAGE. The proteins in the gel were transferred onto 0.45-μm-pore polyvinylidene difluoride (PVDF) membranes and detected with specific or tagged antibodies followed by horseradish peroxidase (HRP)-conjugated secondary antibodies. ImageJ was used to quantify the relative band intensities. The following antibodies were used: GFP-tagged monoclonal antibody (66002-1; Proteintech, IL, USA), Flag-tagged polyclonal antibody (20543-1; Proteintech), β-tubulin polyclonal antibody (10068-1; Proteintech), β-actin polyclonal antibody (20536-1; Proteintech), glyceraldehyde-3-phosphate dehydrogenase (GAPDH) polyclonal antibody (10494-1; Proteintech), and ABHD16A/BAT5 polyclonal antibody (SRP08788; Saierbio, China).

### Construction of swine ABHD16A-overexpressing cells.

The recombinant lentiviral vector with the sABHD16A sequence was constructed by insertion of cDNA sequences of sABHD16A into a lentiviral vector, pCDH-CMV-MCS-EF1-copGFP-T2A-Puro. The primer sequences were as follows: forward (F), 5′-CGGAATTCGCCACCATGGCGAAGCTGCTG-3′; reverse (R), 5′-CGGGATCCCTAGAGGTGCCAGGGCATC-3′. Next, the recombinant lentiviruses were packaged in HEK293T cells by transfection of a recombinant vector with sABHD16A. Eight to 10 h posttransfection, the culture supernatant was removed, cells were washed twice with PBS, and then the medium was replaced with fresh medium. After 48 h, the supernatant was collected and filtered with a 0.45-μm-pore filter. Next, the virus solution was concentrated with polyethylene glycol 8000 (PEG 8000)-NaCl. The empty vector was also packaged into viruses as a control in the same manner. The concentrated recombinant lentiviruses were used to infect PK15 cells at an MOI of 1. Next, 4 μg/mL puromycin was added to the culture medium for cell selection. Postinfection (48 h), the cell culture medium was replaced with the fresh medium every 2 days. The stable monoclonal sABHD16A-expressing cells were obtained through the limited dilution culture method. The expression of sABHD16A of constructed cells was identified by Western blotting.

### Construction of human *abhd16a*^–/–^ cells.

The CRISPR/Cas9 system was used to construct the *abhd16a***^–/–^** cell line. The primers of CRISPR/Cas9 guide RNA (gRNA) targeting the human *abhd16a* gene were designed using the *abhd16a* gene sequence (GenBank accession no. NM_021160.2). The primer sequences are as follows: sgRNA-F, 5′-CACCGTCGCCTTCTTCTACTTGTAC-3′; sgRNA-R, 5′-AAACGTACAAGTAGAAGAAGGCGAC-3′. The following parameters were used for annealing primer oligonucleotides: a boiling water bath for 5 min and naturally cooled to room temperature. Next, the annealing primer was cloned into the pHMG-sgRNA linearized vector. The plasmids of pHMG-sgRNA-sgRNA*abhd16a* were transfected into HEK293 cells and screened with 2 ng/μL puromycin. The monoclonal *abhd16a*^–/–^ cells were obtained using the limiting dilution culture method.

### Construction of ABHD16A mutants.

Three N-terminal truncation (ΔN) mutants of ABHD16A ΔN1/ΔN2/ΔN3 were amplified by PCR using specific primers targeting the 5′-terminal sequence of ΔN1/ΔN2/ΔN3 and 3′-terminal sequence of GFP (Table S1). Next, these were subcloned into pEGFP-N1. The S355A-GFP, Δmotif1-GFP, and Δmotif2-GFP mutants were generated based on the bioinformatics analysis and the principle of fusion PCR. The primers for site mutation were designed and constructed to mutate the target amino acid residues to alanine.

### Acyl-PEGyl exchange gel shift assay.

The acyl-PEGyl exchange gel shift (APEGS) assay was performed using the method described by Percher et al. (57) and Kanadome et al. (58) and modified by our laboratory. In brief, the cell lysate was incubated with tris-(2-carboxyethyl) phosphine (TCEP) and *N*-ethyl maleimide (NEM) at 25°C. All proteins were recovered twice by chloroform-methanol precipitation (CMppt) and dissolved in TEA buffer (4 mM EDTA, 4% SDS). The mixtures described above continued to be incubated with 0.75 M NH_2_OH. The S-palmitate of cysteines was replaced by maleimide-conjugated PEGs (mPEGs) (5 kDa) at 25°C in the TEA buffer (0.2% Triton X-100). The palmitoylation blots of all test samples were detected with monoclonal anti-FLAG M2 through Western blotting. ImageJ was used to calculate the band intensity for quantitative analysis of S-palmitoylated protein.

### Statistical analysis.

All data are presented as the means ± standard error of the mean (SEM). An unpaired two-tailed *t* test was used to determine significant differences between two groups, and one-way analysis of variance (ANOVA) followed by a Tukey’s *post hoc* test was used to evaluate differences between three or more groups. Statistical significance was set as *P* < 0.05 (*, *P* < 0.05; **, *P* < 0.01; ***, *P* < 0.001; ns, not significant). All analyses were performed using GraphPad Prism 8 (GraphPad Software, CA, USA).
