# Supplementary figures and images for: ABHD16A Negatively Regulates the Palmitoylation and Antiviral Function of IFITM Proteins
Source: mBio. 2022 Oct 31;13(6):e02289-22. doi: 10.1128/mbio.02289-22 (PMC9765265; doi:10.1128/mbio.02289-22)

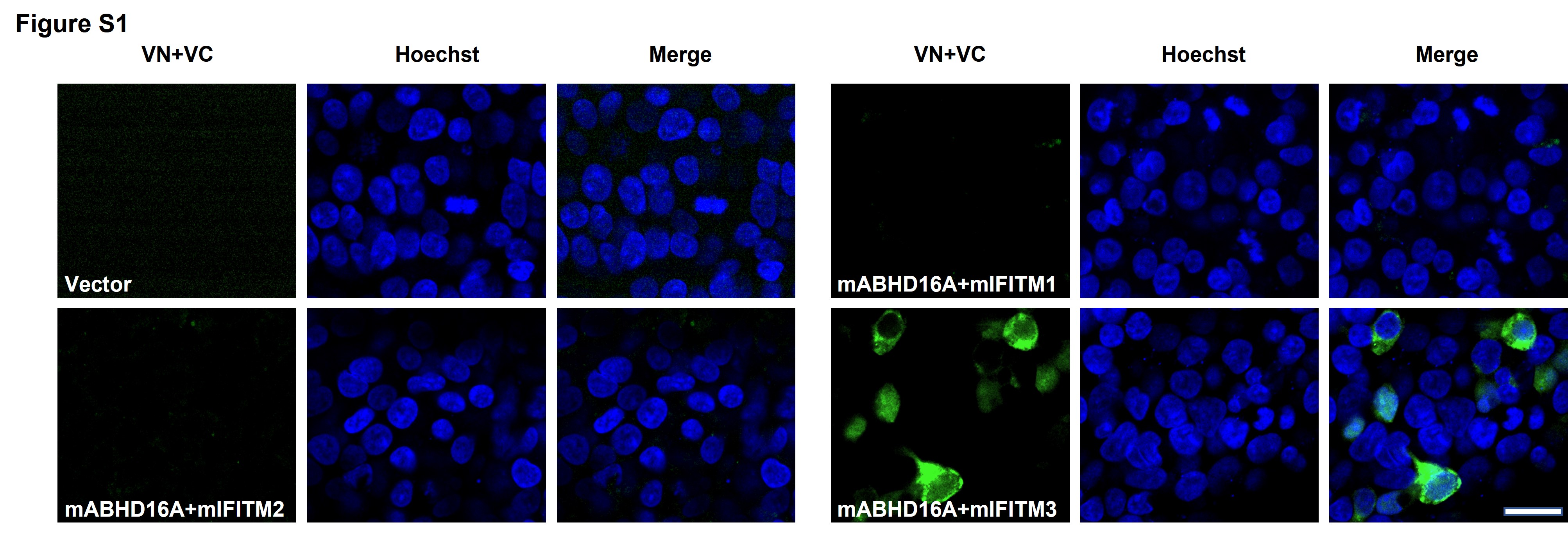

Supplement: FIG S1 [file mbio.02289-22-s0001.tif]

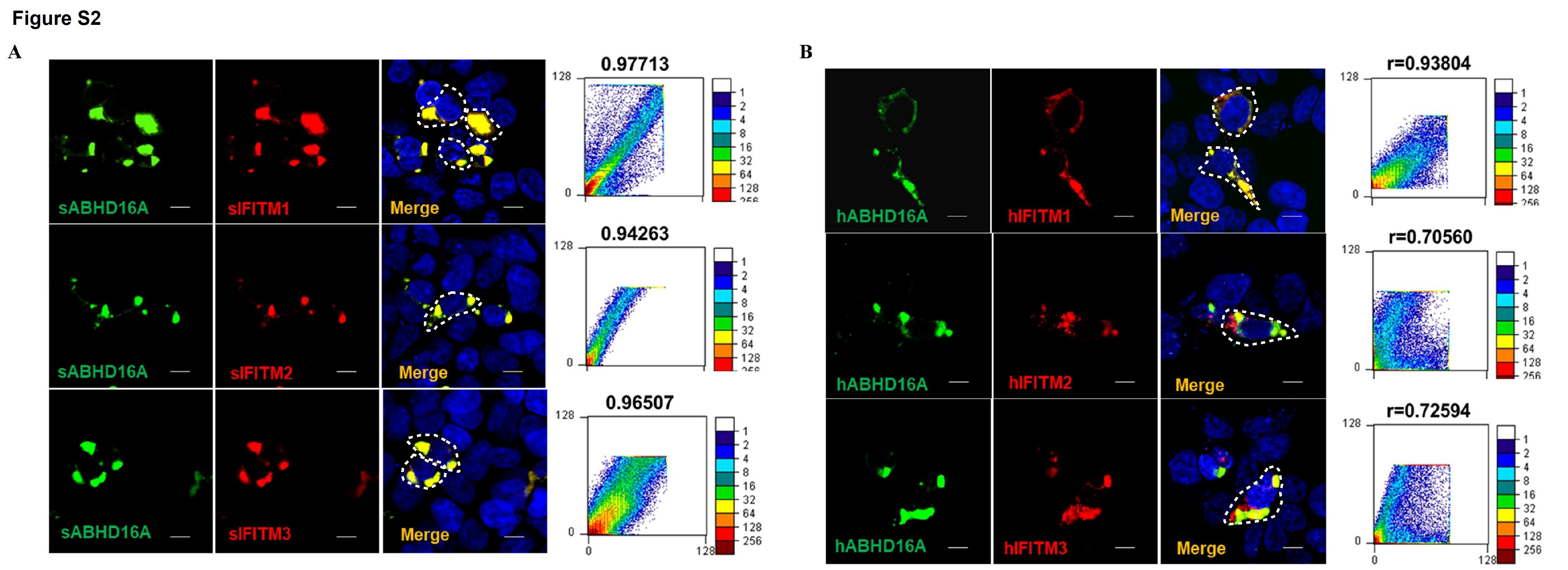

Supplement: FIG S2 [file mbio.02289-22-s0002.tif]

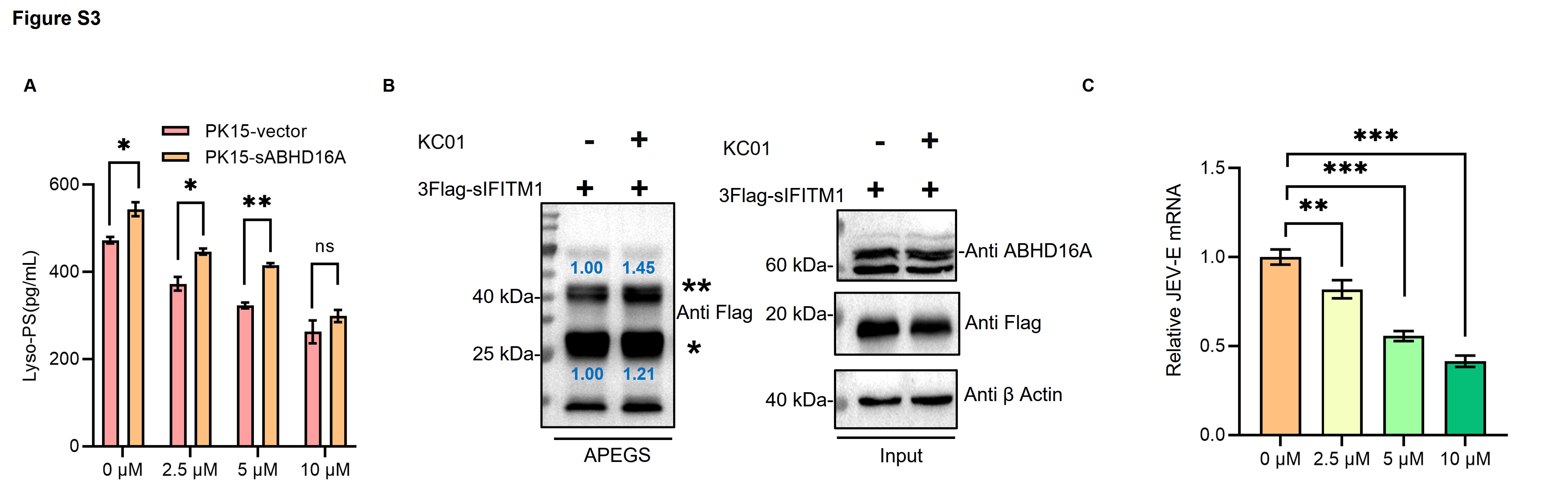

Supplement: FIG S3 [file mbio.02289-22-s0003.tif]
