# Supplementary material for: ABHD16A Negatively Regulates the Palmitoylation and Antiviral Function of IFITM Proteins
Source: mBio. 2022 Oct 31;13(6):e02289-22. doi: 10.1128/mbio.02289-22 (PMC9765265; doi:10.1128/mbio.02289-22)
Supplement: TABLE S1 [file mbio.02289-22-s0005.docx]

**Table S1. The primers for the construction of recombinant vectors**

| gene | vectors | Primer sequence: F (Forward primer), R (Reverse primer) |
| --- | --- | --- |
| *sifitm*1 | pDsRed-monomer-N1 or pGBKT7 | F:5′-CGGAATTCATGATCAAGAGCCAGCAC-3′  R:5′-CGGGATCCCGGTAGCCTCTGTTACTCTTTG-3′ |
|  | pcDNA3.1(+) | F:5′-CGGGATCCATGATCAAGAGCCAGCACGA-3′  R:5′-CGGAATTCCTAGTAGCCTCTGTTACTCT-3′ |
| *sifitm*2 | pDsRed-monomer-N1 or pGBKT7 | F:5′-CGGAATTCATGAACTGCGCTTCCCAGCC-3′  R:5′-CGGGATCCCGGTAGCCTCTGTTACTCTTTG-3′ |
|  | pcDNA3.1(+) | F:5′-CGGGATCCATGAACTGCGCTTCCCAGCC-3′  R:5′-CGGAATTCCTAGTAGCCTCTGTTACTCTTTG-3′ |
| *sifitm*3 | pDsRed-monomer-N1 or pGBKT7 | F:5′- CGGAATTCATGAACTGCGCTTCCCAGCC-3′  R:5′- CGGGATCCCGTAGCCTCTGTAATCCTTTA-3′ |
|  | pcDNA3.1(+) | F:5′- CGGGATCCATGAACTGCGCTTCCCAGCC-3′  R:5′-CGGAATTCCTAGTAGCCTCTGTAATCCTTTA-3′ |
| *sabhd*16a | pEGFP-N1 or pGADT7 | F:5′-CGGAATTCGCCACCATGGCGAAGCTGCTGAGCTG -3′  R:5′-CGGGATCCTCGAGGTGCCAGGGCATCTG-3′ |
|  | pcDNA3.1(+) | F:5′-CGGGATCCGCCACCATGGCGAAGCTGCTGAGCTG -3′  R:5′-CGGAATTCTCGAGGTGCCAGGGCATCTG-3′ |
| *sabhd*16aΔN1 | pEGFP-N1 | F:5′-CGGAATTCGCCACCATGACCCTCCTCAACCGGG-3′ |
| *sabhd*16aΔN2 | pEGFP-N1 | F:5′-CGGAATTCGCCACCATGTGGAACCATCCCGGCT-3′ |
| *sabhd*16aΔN3 | pEGFP-N1 | F:5′-CGGAATTCGCCACCATGATCGGCGGCTTCACTG-3′ |
| *hifitm*1 | pDsRed-monomer-N1 | F:5′-CGGAATTCATGCACAAGGAGGAACA-3′  R:5′-CGGGATCCCTGTAACCCCGTTTTTCCTG-3′ |
|  | pCDNA3.1(+) or pGBKT7 | F:5′-GCGAAGCTTATGCACAAGGAGGAAC-3′  R:5′-CGGAATTCCTAGTAACCCCGTTTTTCCT-3′ |
| *hifitm*2 | pDsRed-monomer-N1 | F:5′-CGGAATTCGCCACCATGAACCACATTGTGCAA-3′  R:5′-CGGGATCCCTTCGCTGGGCCTGGACGACCA-3′ |
|  | pCDNA3.1(+) or pGBKT7 | F:5′-GCGAAGCTTATCTATCGCTGGGCCTGG-3′  R:5′-CGGAATTCGTCACCATGAACCACATT-3′ |
| *hifitm*3 | pDsRed-monomer-N1 | F:5′-CGGAATTCATGAATCACACTGTCCAA-3′  R:5′-CGGGATCCCTTCCATAGGCCTGGAAGA-3′ |
|  | pCDNA3.1(+) or pGBKT7 | F:5′-GCGAAGCTTATGAATCACACTGTCC-3′  R:5′-CGGAATTCCTATCCATAGGCCTGGAA-3′ |
| *habhd*16a | pEGFP-N1 | F:5′-CGGAATTCATGGCGAAGCTGCTG-3′  R:5′-CGGGATCCAGGTGCCAGGGCAT-3′ |
|  | pGADT7 | F:5′-CGAAGCTTATGGCGAAGCTGCTG-3′  R:5′-CGGAATTCCTAGAGGTGCCAGGGCAT-3′ |
| *mifitm*1 | pCDNA3.1(+) | F:5′-CGGGATCCATGCCTAAGGAGCAGCAAGAG-3′  R:5′-CGGAATTCTCATCTAATGGCACAGACA-3′ |
| *mifitm*2 | pCDNA3.1(+) | F:5′-CGGGATCCATGAGCCACAATTCTCAAG-3′  R:5′-CGGAATTCCTAGAATCCAGAATGGGGTG-3′ |
| *mifitm*3 | pCDNA3.1(+) | F:5′-CGGGATCCATGAACCACACTTCTCAAG-3′  R:5′-CGGAATTCTTAAGTGTGAAGGTTTTGAGC-3′ |
