# Supplementary material for: ABHD16A Negatively Regulates the Palmitoylation and Antiviral Function of IFITM Proteins
Source: mBio. 2022 Oct 31;13(6):e02289-22. doi: 10.1128/mbio.02289-22 (PMC9765265; doi:10.1128/mbio.02289-22)
Supplement: TABLE S2 [file mbio.02289-22-s0006.docx]

**Table S2. The primers of qPCR of viral infection experiments**

| Gene type | Gene name | Forward primer 5′ to 3′ | Reverse primer 5′ to 3′ |
| --- | --- | --- | --- |
| Endogenous Gene | *sabhd16a* | TCCTCAACCAGGTCAAGAAGC | GCTGTCCCCCGCCGGTCCAC |
| Envelope protein gene of JEV | *JEV-E* | ACTGACATCTCGACGGTGGC | CTCCCAATCGCTTTACTGGT |
| Nucleocapsid protein gene of VSV | *VSV-N* | GATAGTACCGGAGGATTGACGACTA | TCAAACCATCCGAGCCATTC |
| Reference Gene | *hgapdh* | GACCACAGTCCATGCCATCAC | GCCTGCTTCACCACCTTCTT |
| Reference Gene | *sβ-actin* | AGAGCAAGAGAGGCATCCTG | CACGCAGCTCGTTGTAGAAG |
